# From Widespread Use to Loss of Effectiveness: The Consequences of Inappropriate Azithromycin Prescriptions During the COVID‐19 Pandemic—A Systematic Review and Meta‐Analysis

**DOI:** 10.1155/ijm/8643896

**Published:** 2026-04-27

**Authors:** Beatriz Birelli do Nascimento, Bianca Gianola Belline Silva, Leandro Aparecido de Souza, Jessica Cristina Bilizario Nogueirol Andrade, Fernando de Sá Del Fiol

**Affiliations:** ^1^ Doctoral Program in Pharmaceutical Sciences, University of Sorocaba, São Paulo, Brazil, uniso.br

**Keywords:** azithromycin resistance, macrolides, COVID-19, antimicrobial stewardship, meta-analysis

## Abstract

**Objectives:**

We are aimed at evaluating whether the widespread use of azithromycin during the COVID‐19 pandemic led to a significant increase in bacterial resistance compared with the prepandemic period and estimating the magnitude of this effect through a systematic review and meta‐analysis.

**Methods:**

This systematic review followed the PRISMA 2020 guidelines and Cochrane Handbook (Page,2021). Observational studies published between 2015 and 2025 reporting azithromycin resistance before and after the COVID‐19 pandemic were identified. Odds ratios (ORs) were pooled using a random‐effects model. Methodological quality was assessed using the Newcastle–Ottawa scale.

**Results:**

Eight studies met the eligibility criteria and were included in the quantitative synthesis. The meta‐analysis demonstrated a significant increase in azithromycin resistance in the postpandemic period, with a pooled OR of 2.71 (95% CI: 2.04–3.59). Substantial heterogeneity was observed (*I* ^2^ = 73.5*%*), justifying the use of a random‐effects model.

**Conclusions:**

The findings provide robust evidence that the excessive and largely empirical use of azithromycin during the COVID‐19 pandemic contributed to a global rise in bacterial resistance. Strengthening antimicrobial stewardship policies is essential to preserve the clinical effectiveness of macrolides during future public health emergencies.

**Summary**

• Azithromycin resistance tripled globally after the COVID‐19 pandemic.
• Massive misuse of azithromycin fueled resistance in key bacterial species.
• Odds ratio (OR) of 2.71 confirms a sharp postpandemic rise in resistance.
• Inappropriate prescribing had measurable global microbiological impact.
• The “COVID kit” strategy caused long‐term harm to antimicrobial efficacy.
• Evidence demands urgent action to restore rational antibiotic policies.

## 1. Introduction

In December 2019, health authorities in the city of Wuhan, Hubei Province, China, reported a cluster of pneumonia cases of unknown origin, many of which were epidemiologically linked to a local seafood and live animal market [1, 2]. Subsequent investigations led to the identification of a novel betacoronavirus, genetically distinct from the previously known SARS‐CoV and MERS‐CoV, and provisionally named 2019‐nCoV [1, 3]. The pathogen was isolated from lower respiratory tract samples of affected patients and confirmed through complete genome sequencing and real‐time RT‐PCR testing [3].

The first infected patients presented with typical symptoms of viral respiratory infection, such as fever, cough, and dyspnea, and many required hospitalization, including admission to intensive care units. Clinical and laboratory analyses of these cases revealed high levels of inflammatory markers, and a radiological pattern of bilateral viral pneumonia, similar to that observed in SARS‐CoV infections, raising concerns about the severity and pandemic potential of the new virus [4].

A zoonotic origin was suggested based on the exposure history to the animal market, although the intermediate host between bats, pangolins, and humans has not yet been definitively identified. In the early weeks of 2020, sustained human‐to‐human transmission was documented, including among family members and healthcare professionals, indicating a significant potential for global spread. [3]. Initial epidemiological estimates indicated a basic reproduction number (R_0_) between 2 and 3, suggesting rapid transmission of the agent in susceptible populations. Since then, the virus, later renamed SARS‐CoV‐2 has spread globally, marking the beginning of a pandemic that would redefine clinical practices, public policies, and epidemiological surveillance standards worldwide [1, 2].

In the absence of effective treatments and available vaccines, the scientific community and the global population faced a period of uncertainty, fear, and anxiety. In this context, French microbiologist Didier Raoult, from the Institut Méditerranée Infection in Marseille, published studies suggesting that the combination of hydroxychloroquine, zinc, and azithromycin could reduce the viral load in patients with COVID‐19 [5, 6]. Although based on limited samples and questionable methodologies, these publications received widespread attention and influenced clinical practices in several countries.

The widespread adoption of this protocol, even in the absence of robust scientific evidence, led to a significant increase in azithromycin consumption worldwide [7] [8] [9]. In Brazil, for example, studies indicated that azithromycin sales increased from 1.40 to 3.53 defined daily doses per 1000 inhabitants between February and July 2020 [10]. In addition, there was an increase in dental and veterinary prescriptions of the antibiotic, possibly intended for human use. [11, 12]. For veterinary prescriptions, the increase between 2014 (prepandemic) and 2021 (during the pandemic) was approximately 212%, raising azithromycin’s market share in the veterinary sector from 4% to 8%, in other words, doubling the number of veterinary prescriptions for azithromycin.

The indiscriminate use of the drug during the pandemic raised concerns about the development of antimicrobial resistance, as research has shown a significant increase in microbial resistance to azithromycin in the postpandemic period^1^. For example, a study conducted in Kenya revealed that resistance of *Escherichia coli* and *Shigella* spp. to the antibiotic increased from 6.3% before the pandemic to 40.4% after the pandemic [13].

Several studies have shown that the indiscriminate use of antibiotics in certain regions or countries is directly associated with increased rates of bacterial resistance, often driven by the selection and dissemination of resistance genes that compromise the effectiveness of these drugs. Prolonged and excessive use of antibiotics exerts selective pressure on microorganisms, favoring resistant strains and facilitating the horizontal transfer of genes through mobile elements such as plasmids and integrons [14] [15]. In countries with high antimicrobial consumption, such as India, China, and Brazil, a concerning increase in multidrug‐resistant strains has been observed, especially among community‐ and hospital‐acquired pathogens [16].

In the context of the COVID‐19 pandemic, azithromycin was widely prescribed, often without clinical evidence of concomitant bacterial infection, leading to an unprecedented use of this macrolide [17]. Such a scenario, although understandable given the therapeutic uncertainty at the time, has certainly had and will likely continue to have lasting impacts on the effectiveness of this antibiotic as an antimicrobial agent.

Prepandemic refers to the number of resistant strains over the total number of strains isolated before January 2020. Postpandemic refers to the number of resistant strains over the total number of strains isolated from January 2020 onward.

## 2. Objective

The present study is aimed, through a systematic review followed by meta‐analysis, at determining whether, two years after the end of the international public health emergency, there is consistent evidence that the intensive use of azithromycin compromised its antibacterial activity, especially against species that were previously largely susceptible, using the OR as the measure of effect.

## 3. Methodology

### 3.1. PRISMA Flow Diagram for Study Selection

**Figure 1:**
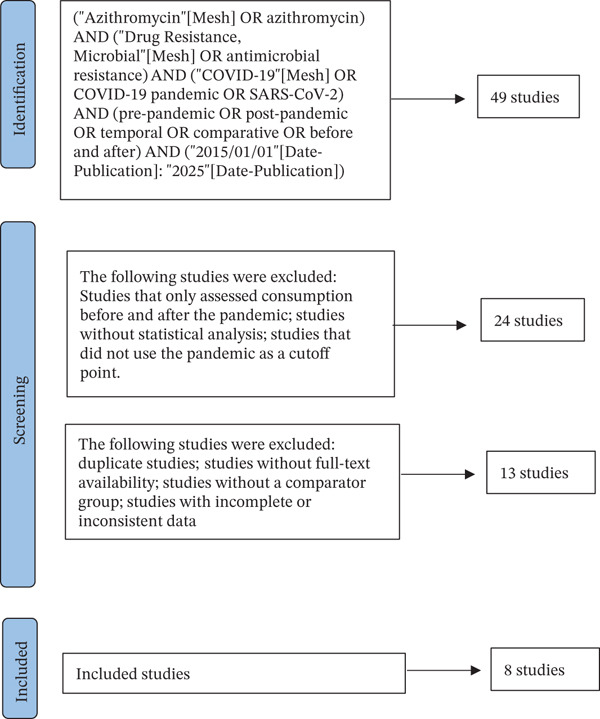
PRISMA 2020 flow diagram illustrating the study selection process. The diagram summarizes the number of records identified, screened, excluded, and the final studies included in the systematic review and meta‐analysis.

The study selection process followed the PRISMA 2020 guidelines and is summarized in Figure 1. A total of records were identified through database searching and hand‐searching. After removing duplicates, the remaining records were screened by title and abstract. Full texts of potentially eligible studies were then assessed according to the predefined inclusion and exclusion criteria. Ultimately, eight studies met all eligibility requirements and were included in the qualitative synthesis and meta‐analysis.

### 3.2. Pico Framework of the Research Question

This systematic review with meta‐analysis was conducted in accordance with the methodological principles established by the *Cochrane Handbook for Systematic Reviews of Interventions*. [18] and reported according to the PRISMA 2020 guidelines (Preferred Reporting Items for Systematic Reviews and Meta‐Analyses) [19].

The research question was developed based on the PICO strategy, which guides the formulation of structured and relevant clinical questions for evidence synthesis.

*P (Population)*: Microorganisms are isolated from human patients subjected to antimicrobial susceptibility testing.

*I (Intervention)*: Clinical use of azithromycin in different contexts and bacterial infections is studied.

*C (Comparison)*: Periods of azithromycin use before the COVID‐19 pandemic versus during and after the pandemic (starting from January 2020) are compared, and.

*O (Outcome)*: Change in bacterial resistance rates to azithromycin are measured using standardized laboratory methods (e.g., disk diffusion, MIC).

### 3.3. Inclusion and Exclusion Criteria

Observational and experimental studies published between 2015 and 2025 were included, provided they presented quantitative data on bacterial resistance to azithromycin in microorganisms isolated from human clinical samples, with a comparison between pre‐ and post–COVID‐19 pandemic periods. Only studies that used standardized susceptibility testing methods (such as disk diffusion, MIC, or automated tests) and reported the number of azithromycin‐sensitive and ‐resistant strains were considered. Articles written in English, Portuguese, or Spanish were accepted.

Studies conducted in veterinary or environmental settings, review articles, opinion pieces, and publications without original data were excluded. Studies that did not distinguish between collection periods (pre‐ and postpandemic), that did not employ recognized resistance analysis methods, or that presented incomplete or duplicate data were also excluded.

### 3.4. Data Extraction

Data extraction was performed using a structured Microsoft Excel spreadsheet in which essential information from each included study was organized. Extracted data included name of the first author, year of publication, and country where the study was conducted; the methodological design adopted (e.g., cross‐sectional study and retrospective cohort); the period of microbiological sample collection, clearly distinguishing the prepandemic period (up to December 2019) and the postpandemic period (from January 2020 onward); the total number of bacterial samples analyzed; the bacterial species isolated in each period; and the levels of resistance to azithromycin reported, expressed either as the percentage of resistant strains or as minimum inhibitory concentration (MIC) values, according to the methodology used in the original studies.

Data extraction was performed independently by two reviewers. Both reviewers extracted all predefined variables using a standardized spreadsheet. Any discrepancies identified during the extraction process were resolved by consensus between the two reviewers, without the need for a third evaluator.

### 3.5. Methodological Quality Assessment of Observational Studies

The methodological quality of the observational studies included in this systematic review was assessed using the Newcastle–Ottawa Scale (NOS) [20], a widely used tool for evaluating the risk of bias in nonrandomized studies. The scale assesses three main domains: (i) selection of study groups (maximum of four stars), (ii) comparability between groups (maximum of two stars), and (iii) outcome or exposure assessment (maximum of three stars), totaling up to nine stars. Each study was rated based on these criteria, and those scoring between seven and nine stars were considered to have a low risk of bias, whereas lower scores indicated moderate risk. This approach was adopted in accordance with the methodological guidelines proposed by Wells et al. in the official document from the University of Ottawa, which is widely referenced in systematic literature reviews [20].

### 3.6. Statistical Analysis

The statistical analysis of the data was conducted through a meta‐analysis, using the OR as the effect measure, which is appropriate for comparing the proportion of bacterial strains resistant to azithromycin between the pre‐ and post–COVID‐19 pandemic periods. Due to the expected variability among the included studies, in terms of study location, analysis method, and bacterial species evaluated, a random‐effects model was adopted, as recommended by the *Cochrane Handbook for Systematic Reviews of Interventions*. [21]. This model was chosen as it better accommodates clinical and methodological heterogeneity. The consistency of results across studies was assessed using the *I* ^2^ heterogeneity test, with values above 75% considered high. To explore the robustness of the findings, sensitivity analyses were also conducted by selectively excluding studies with greater weight or moderate risk of bias. The results were presented using forest plots, showing the combined ORs and their respective 95% confidence intervals, in accordance with the PRISMA 2020 guidelines [19] and the statistical recommendations of Deeks et al. [18].

## 4. Results

Table 1 revealed a marked increase in azithromycin resistance among different microorganisms and geographic regions following the onset of the COVID‐19 pandemic. Several clinically relevant pathogens, such as *Staphylococcus aureus*, *E. coli*, *Shigella* spp., *Haemophilus* spp., and *Bordetella pertussis*, showed consistent rises in resistance rates. Notably, data from Zondag et al. [22] identified a significant increase in *Neisseria gonorrhoeae* resistance during the lockdown period in the Netherlands. These findings are in line with recent literature warnings, such as those by Rawson et al. [29] and Hsu [17], who warned about the risk of a new wave of antimicrobial resistance resulting from the massive and often unjustified use of antibiotics in patients with COVID‐19, even in the absence of confirmed bacterial infection.

**Table 1:** Microorganisms tested for azithromycin susceptibility in various countries before and after the pandemic.

**Author (year)**	**Microorganism**	**Location**	**Prepandemic resistance**	**Postpandemic resistance**	**Significant increase**
Zondag et al. (2023) [22]	*Neisseria gonorrhoeae*	Netherlands	Low	High (during the lockdown)	Yes
Lopez‐Jácome et al. (2022) [23]	*Staphylococcus aureus*	Mexico	25.7%	42.8%	Yes
Ahmad et al. (2025) [24]	*Haemophilus* spp.	Global	0.7%	12.6%	Yes
Fallah et al. (2024) [25]	Gram‐positives (*S. aureus*, CNS)	Iran	Moderate	High (CNS 61,5%, *S. aureus* 40%)	Yes
Ivanova et al. (2024) [26]	*E. coli* and *Salmonella* spp.	Europe	Moderate	High	Yes
Odundo et al. (2024) [13]	*E. coli* and *Shigella* spp.	Kenya	6.3%	40.4%	Yes
Taleb et al. (2023) [27]	Various (not specified)	Palestine	47.9%	63.4%	Yes
Alhusaynat (2024) [28]	*Streptococcus* and *Staphylococcus* spp.	Iraq	48%	70.3%	Yes

Regarding the methodological quality of the included studies, as shown in Table 2, most articles received high scores on the NOS [20] and were classified as having a low risk of bias. This predominance of methodologically sound studies strengthens the robustness of the results obtained in the quantitative synthesis and ensures greater reliability of the meta‐analysis conclusions, as recommended by the Cochrane Handbook for Systematic Reviews of Interventions and the PRISMA 2020 guidelines.

**Table 2:** Assessment of the methodological quality of each study according to the Newcastle–Ottawa scale (NOS).

**Study**	**Selection (★)**	**Comparability (★)**	**Outcome assessment (★)**	**Total (★)**	**Risk of bias**
Zondag et al. (2023) [22]	**★★★★**	**★★**	**★★★**	9	Low
Lopez‐Jácome et al. (2022) [23]	**★★★**	**★★**	**★★★**	8	Low
Ahmad et al. (2025) [24]	**★★★★**	**★★**	**★★★**	9	Low
Fallah et al. (2024) [25]	**★★★**	**★★**	**★★★**	8	Low
Ivanova et al. (2024) [26]	**★★★★**	**★★**	**★★★**	9	Low
Odundo et al. (2024) [13]	**★★★★**	**★★**	**★★★**	9	Low
Taleb et al. (2023) [27]	**★★★★**	**★**	**★★★**	8	Low
Alhusaynat (2024) [28]	**★★★★**	**★**	**★★★**	8	Low

**Figure 2:**
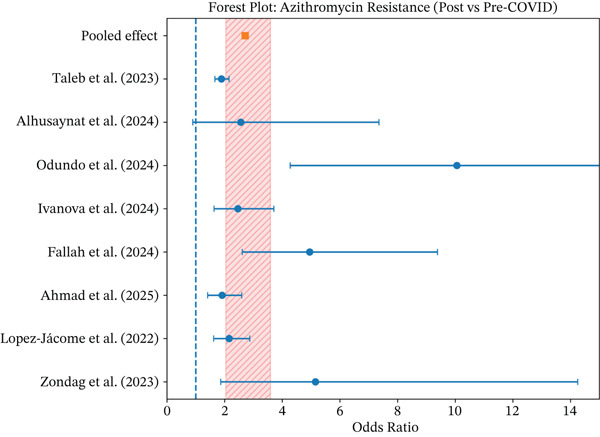
Forest plot of the studies evaluated pre‐ and postpandemic.

Table 3, together with Figure 2 (forest plot), provides robust statistical evidence of an increase in azithromycin resistance in the postpandemic period. The meta‐analysis of eight eligible studies yielded a pooled OR of 2.71 (95% CI: 2.04–3.59), indicating that the odds of bacterial resistance to azithromycin were nearly three times higher after the onset of the COVID‐19 pandemic. This association was statistically significant, with the confidence interval clearly excluding the null value.

**Table 3:** Meta‐analysis (odds ratio and CI 95%) for each evaluated study, including the number of total resistant strains (prepandemic) and the number of resistant and total strains (postpandemic).

**Study**	**Pre (resistant/total)**	**Post (resistant/total)**	**OR (95% CI)**
Zondag et al. (2023) [22]	5/181	18/141	5.15 (1.86–14.25)
Lopez‐Jácome et al. (2022) [23]	106/412	201/470	2.16 (1.62–2.87)
Ahmad et al. (2025) [24]	71/1013	127/1008	1.91 (1.41–2.59)
Fallah et al. (2024) [25]	18/92	59/108	4.95 (2.61–9.38)
Ivanova et al. (2024) [26]	32/345	133/662	2.46 (1.62–3.70)
Odundo et al. (2024) [13]	7/111	44/109	10.06 (4.25–23.68)
Taleb et al. (2023) [27]	977/2039	1160/1827	1.89 (1.71–2.13)
Alhusaynat et al. (2024) [28]	12/25	26/37	2.56 (1.08–6.06)
Combined effect (random‐effects model)			2.71 (2.04–3.59)

Individual studies consistently demonstrated increased resistance across diverse settings and microorganisms. Particularly high effect sizes were observed in Odundo et al. [13], which reported an OR greater than 10, suggesting a pronounced impact in specific populations and epidemiological contexts. Other studies, including Zondag et al. [22], Fallah et al. [25], and Ivanova et al. [26], also reported substantial increases in resistance, reinforcing the consistency of the observed trend.

Substantial heterogeneity was detected among studies (*Q* = 26.40; *d* *f* = 7; *I* ^2^ = 73.5*%*), reflecting differences in study design, geographic regions, bacterial species, and healthcare settings. Given this variability, the use of a random‐effects model was methodologically appropriate and ensured a conservative and reliable pooled estimate.

## 5. Discussion

This systematic review and meta‐analysis demonstrates a consistent and statistically significant increase in bacterial resistance to azithromycin in the post–COVID‐19 period. Based on aggregated data from eight methodologically robust studies, the likelihood of resistance was almost threefold higher following the pandemic, underscoring the microbiological consequences of widespread and often inappropriate antimicrobial use during this global health crisis.

The magnitude and consistency of the observed effect across heterogeneous settings suggest that the increase in resistance is not confined to a single region, microorganism, or clinical context. Studies such as Ahmad et al. [24] and Ivanova et al. [26], which included large and geographically diverse datasets, support the hypothesis that excessive azithromycin use during the pandemic exerted substantial selective pressure on bacterial populations. Similarly, high effect estimates reported in specific contexts, as observed by Odundo et al. [13], highlight how local prescribing practices and healthcare infrastructure may amplify resistance development.

The substantial heterogeneity identified in this meta‐analysis is not unexpected. Variations in bacterial species, baseline resistance levels, diagnostic capacity, and stewardship policies across countries likely contributed to the observed variability. Nevertheless, despite this heterogeneity, all included studies showed an increase in resistance in the postpandemic period, lending robustness to the overall conclusion.

These findings align with a growing body of literature documenting the excessive and frequently unjustified use of antibiotics during the COVID‐19 pandemic. Langford et al. [30] reported that approximately 75% of hospitalized COVID‐19 patients received antibiotics, despite confirmed bacterial coinfection rates below 10%, illustrating the scale of empirical prescribing. Similarly, Calderón‐Parra et al. [31] demonstrated that a significant proportion of antibiotic prescriptions in COVID‐19 patients were inappropriate and driven by nonspecific clinical findings rather than microbiological evidence.

In some countries, the situation was further exacerbated by political and regulatory factors. In Brazil, for example, azithromycin became a core component of the so‐called “COVID kit,” a combination of drugs promoted for early or preventive treatment despite the absence of scientific support. As described by Caetano et al. [32], this practice was reinforced by misinformation and political endorsement, leading to marked increases in national azithromycin consumption. Comparable patterns were observed in other settings, including Italy, where Ferrara and Albano [33] documented uncontrolled prescribing driven by social pressure and misinformation, resulting in drug shortages and compromised rational use.

Although stewardship efforts and accumulating evidence later led to a decline in empirical azithromycin use, the microbiological impact appears to persist. Studies such as Sili et al. [34] showed a gradual reduction in azithromycin prescribing as understanding of bacterial coinfection rates improved; however, the selective pressure exerted during earlier pandemic phases may have already altered resistance patterns. Moreover, evidence from Antonazzo et al. [35] indicates that community use of azithromycin failed to improve key clinical outcomes while potentially increasing adverse events, further questioning its widespread empirical use.

## 6. Conclusions

This systematic review and meta‐analysis provides compelling quantitative evidence that the widespread and largely inappropriate use of azithromycin during the COVID‐19 pandemic was associated with a significant increase in bacterial resistance. The nearly threefold rise in resistance observed across diverse settings highlights the vulnerability of antimicrobial effectiveness during periods of public health uncertainty, when empirical prescribing practices tend to intensify. These findings emphasize the urgent need to strengthen antimicrobial stewardship programs, ensure adherence to evidence‐based prescribing guidelines, and safeguard the rational use of antibiotics during future health emergencies. Without decisive and sustained interventions, the clinical utility of azithromycin and potentially other critical antimicrobials may be further compromised, limiting treatment options for common respiratory, enteric, and sexually transmitted infections. Taken together, the findings underscore the importance of adhering to clinical guidelines and scientific evidence when managing infectious diseases, especially during health crises. The reliance on treatments driven by misinformation, political influence, or public pressure can lead to lasting and potentially irreversible consequences for antimicrobial effectiveness. The observed decline in azithromycin susceptibility serves as a clear indication that future public health policies must prioritize scientific rigor and transparent communication. Continued research will be essential to confirm these trends and to assess the broader microbiological impact of pandemic‐era prescribing behaviors, but the present evidence already offers a stark reminder of the risks posed by deviating from evidence‐based practice.

## 7. Limitations of the Present Study

Although the strengths of this study, such as the methodological rigor, adherence to PRISMA 2020 standards, and the use of high‐quality observational data support the robustness of the overall conclusions, several limitations should also be acknowledged. First, although all included studies distinguished between pre‐ and postpandemic periods, the precise boundaries of these intervals varied among authors, potentially introducing temporal heterogeneity. Additionally, despite the use of standardized laboratory methods, technical differences across microbiology laboratories, such as variations in susceptibility testing protocols, bacterial strains evaluated, and interpretive criteria could not be fully controlled. Further limitation relates to the geographical breadth of the included studies which, while enhancing the scope and external validity of the review, may reduce the applicability of the findings to specific regional contexts. Future research incorporating longitudinal designs, methodological harmonization, and targeted subgroup analyses may help generate even more precise and region‐specific evidence.

## Conflicts of Interest

The authors declare no conflicts of interest.

## Funding

No funding was received for this manuscript.

## Endnotes

^1^Prepandemic refers to the number of resistant strains over the total number of strains isolated before January 2020. Postpandemic refers to the number of resistant strains over the total number of strains isolated from January 2020 onward.

## Data Availability

The data that support the findings of this study are available from the corresponding author upon reasonable request.
